# Introduction

**DOI:** 10.1038/sj.bjc.6601475

**Published:** 2003-12-10

**Authors:** U Gatzemeier

**Affiliations:** 1 Department of Thoracic Oncology, Krankenhaus Großhansdorf, Wohrendamm 80, Großhansdorf 22927, Germany

Globally, lung cancer is the leading cause of cancer-related mortality in both men and women (Ferlay *et al*, 2000), with non-small-cell lung cancer (NSCLC) accounting for 80% of these cases. Many patients present with advanced/metastatic disease. Despite aggressive surgical or chemotherapeutic intervention, the prognosis of patients diagnosed with NSCLC remains poor, with an overall cure rate of <15% (Sridhar *et al*, 2003). Therefore, new safe and effective treatments for NSCLC are urgently needed.

Aberrant cell signalling by receptor tyrosine kinases such as the epidermal growth factor receptor (EGFR) is known to play a critical role in the development and progression of cancer (Ullrich *et al*, 1984; Zwick *et al*, 2001). The EGFR pathway is highly expressed in a variety of solid tumours, including NSCLC, and has been implicated in tumorigenesis through its effects upon cell-cycle progression, apoptosis, angiogenesis, tumour-cell motility and metastasis (Ciardiello and Tortora, 2001; Salomon and Gullick, 2001; Arteaga, 2002; Bunn Jr and Franklin, 2002). As EGFR expression correlates with poor prognosis, disease progression and resistance to chemotherapy (Baselga, 2002; Wells, 2000), it has been identified as a potential therapeutic target in the treatment of cancer.

Gefitinib (‘Iressa’, ZD1839) is the first of a new class of EGFR tyrosine kinase inhibitors and, as such, physicians and patients are taking a great interest in its clinical profile and development. Two Phase II monotherapy trials (‘Iressa’ Dose Evaluation in Advanced Lung cancer (IDEAL) 1 and 2) have reported unprecedented antitumour activity and symptom relief in pretreated patients with advanced/metastatic NSCLC (Fukuoka *et al*, 2003); approximately 40% of patients experienced objective responses and stable disease accompanied by improvement in disease-related symptoms and 30% of patients survived for 1 year. The IDEAL trials underpinned the current use of gefitinib in clinical practice and, as of September 2003, gefitinib had been administered to approximately 90 000 patients worldwide. The only FDA-approved option for use in patients with NSCLC that has failed both platinum-based and docetaxel chemotherapy in the USA, gefitinib is also approved for use in previously treated patients in several other countries, including Japan and Australia. Our thirst for new knowledge of how best to use this novel targeted agent remains unquenched, and data from the real-life use of gefitinib can provide invaluable insight into the clinical application of gefitinib in a wide variety of settings.

The ‘Iressa’ Expanded Access Programme (EAP) enables patients to receive 250 mg day^−1^ gefitinib if they are ineligible for clinical trials or have no other treatment options available. To date (September 2003), approximately 40 000 generally heavily pretreated patients with advanced NSCLC, in 73 countries, have received gefitinib on a compassionate-use basis via the EAP. Thus, the EAP provides a wealth of real-life experience of using gefitinib in elderly patients, patients with poor performance status and those with brain metastases. It is important that the experience of using gefitinib in the EAP is shared between all physicians who are striving to provide the best level of care for their patients, including those who are not necessarily part of the EAP.

In June 2003, the ‘Iressa’ Clinical Experience (ICE) meeting was held in Madrid, Spain, and provided a unique opportunity for 150 EAP investigators to disseminate the real-life experience of gefitinib generated through EAP usage. The sharing of case reports and series by EAP physicians generated an overall (rather than individual) perception of the efficacy, safety and quality-of-life impact of gefitinib. Unique insights into the clinical use of gefitinib were gained from the meeting, some of them unexpected. In order to disseminate this knowledge to all physicians, the data presented at the ICE meeting have been used to describe the concept of assessing clinical benefit, the tolerability, the treatment of patients with brain metastases and the treatment of elderly and unfit patients, using gefitinib in a real-life setting. These data will provide physicians with invaluable insight into the clinical application of gefitinib in a wide variety of settings, and enable them to provide the best level of care for their patients.
